# Classification of tumor types using XGBoost machine learning model: a vector space transformation of genomic alterations

**DOI:** 10.1186/s12967-023-04720-4

**Published:** 2023-11-21

**Authors:** Veronica Zelli, Andrea Manno, Chiara Compagnoni, Rasheed Oyewole Ibraheem, Francesca Zazzeroni, Edoardo Alesse, Fabrizio Rossi, Claudio Arbib, Alessandra Tessitore

**Affiliations:** 1 Department of Biotechnological and Applied Clinical Sciences, University of L’Aquila, 67100 L’Aquila, Italy; 2 Center for Molecular Diagnostics and Advanced Therapies, University of L’Aquila, Via Petrini, 67100 L’Aquila, Italy; 3 Department of Information Engineering, Computer Science and Mathematics, Center of Excellence DEWS, University of L’Aquila, 67100 L’Aquila, Italy

**Keywords:** Machine learning, XGBoost classifier models, Vector Space Transformation, Genomic Alterations, Tumors, Cancer diagnosis

## Abstract

**Background:**

Machine learning (ML) represents a powerful tool to capture relationships between molecular alterations and cancer types and to extract biological information. Here, we developed a plain ML model aimed at distinguishing cancer types based on genetic lesions, providing an additional tool to improve cancer diagnosis, particularly for tumors of unknown origin.

**Methods:**

TCGA data from 9,927 samples spanning 32 different cancer types were downloaded from cBioportal. A vector space model type data transformation technique was designed to build consistently homogeneous new datasets containing, as predictive features, calls for somatic point mutations and copy number variations at chromosome arm-level, thus allowing the use of the XGBoost classifier models. Considering the imbalance in the dataset, due to large difference in the number of cases for each tumor, two preprocessing strategies were considered: i) setting a percentage cut-off threshold to remove less represented cancer types, ii) dividing cancer types into different groups based on biological criteria and training a specific XGBoost model for each of them. The performance of all trained models was mainly assessed by the out-of-sample balanced accuracy (BACC) and the AUC scores.

**Results:**

The XGBoost classifier achieved the best performance (BACC 77%; AUC 97%) on a dataset containing the 10 most represented tumor types. Moreover, dividing the 18 most represented cancers into three different groups (endocrine-related carcinomas, other carcinomas and other cancers),such analysis models achieved 78%, 71% and 86% BACC, respectively, with AUC scores greater than 96%. In addition, the model capable of linking each group to a specific cancer type reached 81% BACC and 94% AUC. Overall, the diagnostic potential of our model was comparable/higher with respect to others already described in literature and based on similar molecular data and ML approaches.

**Conclusions:**

A boosted ML approach able to accurately discriminate different cancer types was developed. The methodology builds datasets simpler and more interpretable than the original data, while keeping enough information to accurately train standard ML models without resorting to sophisticated Deep Learning architectures. In combination with histopathological examinations, this approach could improve cancer diagnosis by using specific DNA alterations, processed by a replicable and easy-to-use automated technology. The study encourages new investigations which could further increase the classifier’s performance, for example by considering more features and dividing tumors into their main molecular subtypes.

**Supplementary Information:**

The online version contains supplementary material available at 10.1186/s12967-023-04720-4.

## Background

Cancer is the second cause of death worldwide after hearth disease, accounting for more than 9 million deaths in 2018 [1]. In the last decades, scientists explored molecular mechanisms at the base of this disease and discovered that genetic features of tumors are crucial to ensure accurate diagnosis and effective therapies. Later, advances in high-throughput technologies allowed more comprehensive exploration of both genetic and epigenetic landscape of cancer. Among them, next generation sequencing (NGS) provided an unprecedented powerful tool to analyze and understand the complexity of cancer genomes. The Cancer Genome Atlas (TCGA) program started fifteen years ago, and more than 20,000 molecularly characterized primary tumors, and matched normal samples, spanning 33 cancer types are now available to the scientific community, providing an extraordinary resource for deepening and further shedding light on tumor complexity. This large collection of datasets (genomic, transcriptomic, epigenomic data), profiled on multiple technological platforms, strongly reinforces the opportunity not only to improve the molecular alterations’ understanding in human cancers, but also to refine the ability to diagnose, treat and prevent cancer in the era of precision medicine [2]. Several studies already underlined the importance of using molecular data to distinguish tumor subtypes [3–7], and now the increase of available data has no previous for systematically analyzing differences and similarities between tumors, based on their genetic and epigenetic traits. It is known that the complex framework of somatic alterations detected in cancer is typically the result of a relatively small number of functional oncogenic alterations (driver mutations), overcame by non-functional alterations (passenger events) which do not substantially contribute to cancer initiation and progression [8, 9]. In this context, the low “signal to noise” ratio between the number of functional and non-functional events is one of the major challenges for data mining and analysis.

More recently, the results of several studies, focused on cancer-type identification, highlighted different patterns of genomic alterations which seem to indicate that tumors originating in the same organ or tissue can vary substantially in terms of lesions [10]. On the other hand, similar patterns can be observed in tumors from tissues of different origin [11]. These phenomena, described as intra-cancer heterogeneity and cross-cancer similarity, represent both a clinical challenge and an opportunity to design, in the future, new diagnostic and therapeutic protocols based on genomic traits of tumors [2]. Notably, aimed at potential applications at the clinical level as well, it would be of great interest to identify a method able to distinguish different tumor subtypes. Consequently, several studies, based on gene expression profiling, have tried to differentiate a relatively small number of cancer types or subtypes, by taking advantage of on-line available datasets [12, 13]. In this scenario, in addition to the development of an effective and reliable approach to distinguish and categorize different types of tumors, it would be very relevant to generate a novel practical cancer diagnostic tool, ideally able to use as few distinctive molecular features as possible.

In the last decades Machine learning (ML) technologies [14] allowed us to handle these complex and high-dimensional cancer genome data, by constructing reliable and easy-to-use automated diagnostic tools for clinical applications [15, 16], thus extracting new information on relationships between molecular alterations and human cancers [17, 18]. Several studies described the applicability of ML models to predict primary sites of cancers from unknown primary tumor [9, 19, 20], to distinguish cancer types and normal tissues [21–23] or also to determine molecular drivers or multi-omics predictor of resistance/response to therapeutic treatments [24, 25]. In addition, ad-hoc replicable feature extraction methods can be used to transform the original genes’ alterations raw data into a structured usable dataset. The latter can feed supervised learning architectures [14] to generate classification models which are able to accurately distinguish multiple cancer types based on the transformed data. Feature selection/ranking algorithms can reduce the complexity of the dataset to improve the accuracy of the trained models and can provide insights on biological mechanisms at the base of cancer.

The aim of this study was to develop a ML approach able to distinguish cancer types with high accuracy, based on somatic point mutations (SPMs) calls and copy number variations (CNVs). At the translational level, this model could improve cancer diagnosis by using specific tumor DNA alterations as cancer diagnostic tools, embedded in a replicable easy-to-use automated technology.

Furthermore, by highlighting differences as well as similarities among tumors, according to gene mutations and copy number alterations rates, further insights into the characterization of the molecular landscape of different tumors can be obtained, thus increasing our understanding of cancer heterogeneity.

## Methods

### Feature extraction strategy and dataset construction

Molecular data, including SPMs and CNVs, of 10,768 samples spanning 32 different cancer types were downloaded from cBioportal (https://www.cbioportal.org/). Most were primary tumors, except for skin cutaneous melanoma, 82% of which were metastases. This dataset derived from TCGA PanCancer Atlas project (https://www.cell.com/pb-assets/consortium/pancanceratlas/pancani3/index.html), goals of which are to assemble consistent TCGA data across tumor types as well as platforms. Studies we included in our analysis have uniform clinical characteristics, consistent processing and normalization of molecular data and are ideally elaborated for comparative analyses [26]: in this context, we considered the number of calls for SPMs and CNVs at chromosome arm-level as predictive features of cancer types in a supervised ML manner.

Of note, SPMs and CNVs represent the main genomic lesions characterized so far as well as the main types of alterations identified in cancers: variants involving few nucleotides (SPMs), are the most studied somatic alterations in human cancers and can now be detected with high accuracy [8]; on the other hand, large genomic rearrangements, particularly CNVs, although less studied, are also relevant to tumorigenesis, representing the prevalent kind of alteration in some tumor types [27].

We devised a feature extraction technique inspired by Computer Science applications, which produces a homogeneous and highly interpretable dataset, and which is easy to implement in a computer program. This technique is of the Vector Space Model (VSM) type [28, 29], in which rough data are transformed into vectors belonging to a certain geometrical space by counting the number of occurrences of specific elements in the considered data. It is worth mentioning that putting data into a geometrical space allows us to compute distance, and so similarities between samples, and consequently to train accurate ML models. The VSM paradigm is often successfully applied in Natural Language Processing (NLP) under the hood of bag-of-words techniques [30], in which texts are transformed into vectors by counting the number of occurrences of certain words. The bag-of-words VSM approach has a major limitation when applied to NLP: the counting mechanism does not consider the order in which elements (words) are observed, which is relevant from a semantic point of view. The adaptation of the VSM approach to our application does not suffer from the previous drawback, as data is not characterized by a relevant ordering pattern. In particular, the proposed feature extraction consisted of counting, for each sample in the dataset, the occurrences of SPMs and CNVs in the p-arm and q-arm of each chromosome. Below, the adopted VSM feature engineering procedure is briefly described. Samples of the original available data were downloaded separated according to each specific tumor type. Assume we have a set *t* = *1,…,T* of tumor types. For each tumor type *t*, two datasets were available: one for SPMs (say SPM[*t*]*)* and one for CNVs (say CNV[*t*]).

Concerning a generic SPM[*t*], it contains data from a set *i* = *1,…,I*^***t***^ of tumor samples, each associated to generally multiple records. Each record corresponds to a single SPM observed for a sample *i*, and reports: the bar code identifier associated to sample *i*, the chromosome where the SPM is detected, and the starting and ending position of the SPM in such chromosome. Notice that the specific p or q arm of the chromosome where the SPM is located can be directly retrieved from these positions. Single Nucleotide Polymorphisms (SNPs), Deletions (DELs), Insertions (INSs) and Oligo Nucleotide Polymorphisms (ONPs) were considered as SPMs. An example of a SPM[*t*] dataset for a generic tumor type *t* is depicted in Additional file 1: Figure S1.

The structure of a given CNV[*t*] is similar to the SPM[*t*] one, reporting for each record: the sample identifier, the chromosome associated to the CNV, the starting and ending position, and the segment mean of the CNV (see e.g. Additional file 1: Figure S2). From the segment mean value, we computed the type of CNV as: Deletions (DLTs) for values ≤ -0.3, Shallow-Deletions (SHDs) for values > -0.3 and ≤ -0.1, Gains (GANs) for values > 0.1 and ≤ 0.3, and Amplifications (AMPs) for values > 0.3. An example of a CNV[*t*] dataset for a generic tumor type *t* is depicted in Additional file 1: Figure S2.

For each tumor type *t*, for each tumor sample *i*, and for both the SPM[*t*] and CNV[*t*] datasets, the VSM procedure consists in counting the occurrence of, respectively, the four SPMs and the four CNVs, for each of the chromosome arm p and q in 23 chromosome pairs (the Y chromosome is excluded from the analysis). This results in two intermediate datasets, denoted for simplicity as VSM-SPM[*t*] and VSM-CNV[*t*]. Then VSM-SPM[*t*] and VSM-CNV[*t*] are merged based on the tumor sample identifiers. This generates, for each tumor type, a VSM-SPM-CNV[*t*] unified dataset with samples of 368 features (4 from VSM-SPM[*t*] and 4 from VSM-CNV[*t*] for each of the 2 arms of the 23 chromosomes).

Finally, all VSM-SPM-CVN[*t*] datasets built for each tumor type *t* are gathered to generate the final VSM-SPM-CVN dataset of 9,927 samples. Such number of samples is lower than the original one (10,768) because when the VSM-SPM[*t*] and VSM-CNV[*t*] were merged, some samples lacked one of the two types of data, and so they were removed. However, the number of the removed samples is not significant with respect to the dimensionality of the whole dataset. Except for this, no missing values issues were encountered.

The pseudocode of the described VSM data transformation procedure as well as the final dataset on which all experiments have been carried out, are reported in Additional file 1: Figure S3 and Additional file 1: Table S1, respectively .

It is worth mentioning that, at first, one may consider datasets with finer grained resolution levels, by further dividing the chromosome arms into subregions in which to count SPMs and CNVs. Experiments have been carried out in this direction but, on the datasets used, while requiring much larger computational effort, they did not provide improvements with respect to the basic arms-based resolution. Therefore, for the sake of brevity, they were not reported here.

In conclusion, after the feature extraction phase, the dataset included 9,927 samples (some samples were removed due to missing values), each consisting of 368 features and one target representing the cancer type.

### Preprocessing phase and ML methods

The dataset of 9,927 instances appeared highly unbalanced, since some cancer types were characterized by a small number of samples, further complicating the already challenging 32-classes classification problem.

Standard techniques for data imbalance, like under-sampling or over-sampling (see e.g., [31]), are not adequate to deal with the considered case-study. Indeed, under-sampling of the most represented tumor seems to result in significant loss of information, as the samples’ fragmentation into many tumor types implies that the number of samples is not so large also for the most represented ones. On the other hand, over-sampling techniques like SMOTE (see [32]), are known to be not particularly suited for multi-class problems with many classes (like the investigated one), as it may cause overlapping of the samples of different classes.

For the reasons above-mentioned, the following two preprocessing strategies have been considered to address the inherent data imbalance:

i. Setting a percentage cut-off threshold to remove from the analysis all cancer types whose percentage of occurrences in the dataset was below an established threshold (similarly to what done in [9]),
ii. Grouping cancer types into different groups, and training a specific ML model for each of them, to have smaller dimensional classification difficulty and make analysis easier. This strategy requires a prior mechanism to recognize to which group a certain sample belongs. We have implemented this groups’ recognition mechanism by training an additional ML model. So, this strategy can be viewed as a sequential two-phase ML approach: for the first phase, a ML model was trained to recognize to which cancer group a certain sample belongs to, and, in the second phase, for each group a specific ML model was trained to determine the cancer type of that group. Clearly, the previous strategies may show drawbacks. Concerning (i), the removal of less represented cancer types makes the ML problem easier, but reduces the purpose of model applicability. As to (ii), the sequential two-phase ML approach, although reducing data imbalance and facilitating cancer type classification within each group, may combine errors of the second phase (intra-group cancer type classification), and errors of the first phase (group recognition). However, in certain practical cases, the group membership of samples may be known “a priori”, so that the first phase is not really required. Moreover, it has to be noticed that the data imbalance is proper of the dataset considered in this work, but in presence of more balanced ones the proposed feature extraction and ML methodology could be applied without the need of implementing strategies (i) or (ii).

Four types of experiments have been compared:

- training a ML model to classify all the 32 cancer types,
- training a ML model to classify a subset of the most represented cancer types (strategy (i)),
- a sequential two-phase approach to classify all the 32 cancer types, (strategy (ii)).
- a sequential two-phase approach to classify a subset of the most represented cancer types (strategies (i) and (ii)).

In all cases the performance of all the trained models has been determined by dividing the dataset into training and testing sets (respectively the 70% and 30% of the samples), by extracting randomly the samples to be included in the testing set to maintaining the proportion between classes of the whole dataset. Of note, the 80/20 and a 90/10 train-test splits were also evaluated, which returned similar results, so 70/30 was chosen, thus considering a larger and more reliable testing set.

The hyperparameters of the trained models have been carefully determined by a grid-search method with a fivefold cross-validation technique [14] to improve the performance and reduce as much as possible overfitting phenomena.

Many ML methods have been tested, including Multi-layer Perceptrons [33, 34], Support Vector Machines [35, 36], K-Nearest-Neighbors [37] and XGBoost [38, 39]. Tests have been performed also with a Deep Learning architecture, by using Convolutional Neural Networks [40].

The reported results are the ones obtained by XGBoost, as, on all the considered experiments, it achieved by far the best performance, as it reveals to be the best compromise between simplicity of the model and expressive power. The performance of the other tested algorithms above-mentioned is shown in Additional file 1: Table S2.

## Results

### Genomic features extraction

In this study, the number of calls for SPMs and CNVs at chromosome arm-level were considered as predictive features of cancer types. As mentioned above, overall, we obtained a dataset of 9,927 samples, each one made up of 368 features and one target representing the cancer type, for a total of 32 different cancers.

**Fig. 1:**
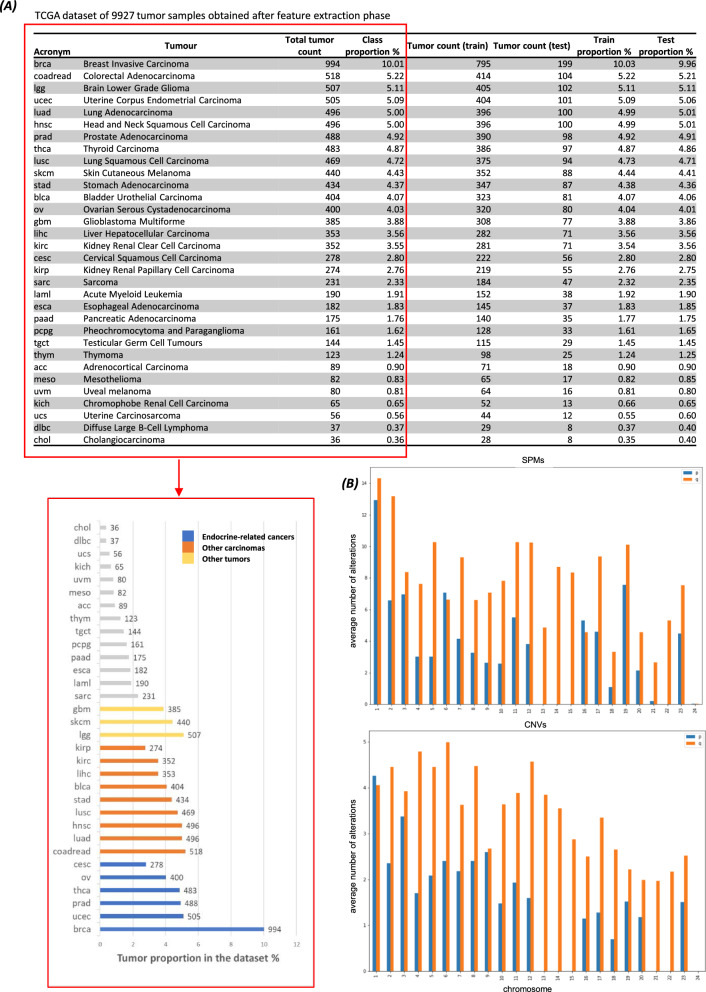
**A** Table showing the dataset used in this study and bar chart displaying the size of each tumor, in terms of number of samples, and the division of the most represented cancer types (N = 18) into groups, based on biological criteria (endocrine-related cancers, other carcinomas and other tumors). The total count for each tumor is reported. **B** Bar chart showing the arithmetic average related to somatic point mutations (SPMs) and copy number variations (CNVs) at chromosome arm-level considering the whole dataset

In Fig. 1A the dataset obtained after feature extraction phase and the descriptive statistics for each tumor type, including number of samples (total tumor count) and relative proportion respect to the whole dataset (class proportion), tumor count used as training and testing sets as well as their corresponding proportion are shown.

Overall, the imbalance in the dataset, due to the different number of samples for each tumor, is well evident. Breast invasive carcinoma (brca) tops the list with 994 samples, while the following eight tumor types [colorectal adenocarcinoma (coadbread), brain lower grade glioma (lgg), uterine corpus endometrial carcinoma (ucec), lung adenocarcinoma (luad), head and neck squamous cell carcinoma (hnsc), prostate adenocarcinoma (prad), thyroid carcinoma (thca) and lung squamous cell carcinoma (lusc)], have a tumor count of approximately 500. Ten tumors [skin cutaneous melanoma (skcm), stomach adenocarcinoma (stad), bladder urothelial carcinoma (blca), ovarian serous cystadenocarcinoma (ov), glioblastoma multiforme (gbm), liver hepatocellular carcinoma (lihc), kidney renal clear cell carcinoma (kirc), cervical squamous cell carcinoma (cesc), kidney renal papillary cell carcinoma (kirp) and sarcoma (sarc)] fall within a range of 230–440 samples; the remaining thirteen tumor types, contribute below 200 in the total number of samples.

Because of this dataset imbalance, pre-processing techniques by considering class grouping (two-phase approach) in a subset of the most represented cancer types was one of the approaches used, including dividing samples into endocrine-related cancers, other carcinomas, and other tumors, for a total of six, nine, and three cancer types in each group respectively, as shown in the bar chart of Fig. 1A.

The distribution of the features, in terms of average number of SPMs and CNVs at chromosome arm-level by considering the whole dataset, is shown in Fig. 1B.

In this work, the combination of the high resolution CNV dataset (Deletion, Shallow-Deletion, Gain and Amplification) and SPM dataset (SNP, DEL, INS and ONP) was considered.

## Performance of the XGBoost classifier models on the preprocessed datasets

Main pre-processing techniques considered in this work were threshold-controlled datasets, use of low resolution cytoBand classification to further divide chromosome arms into subregions and, consequently, increase the number of features, as well as class grouping. The performance of all trained models (reported in Additional file 1: Table S3.) is assessed through the out-of-sample balanced accuracy (BACC) i.e., the average of the accuracy scores obtained on each single class (not suffering from data imbalance), and the AUC scores.

In the following sections, models achieving the best performance are described and the hyperparameter values considered for the grid-search and the selected ones are reported in Additional file 1: Table S4. For these models, also sensitivity, specificity, F1-score and Matthew Correlation Coefficient (MCC) were evaluated, as they may be relevant in case of data imbalance.

### Cut-off threshold experiments

A method of dealing with class imbalance considered in this work was setting threshold for the number of samples corresponding to each cancer type. This technique uses only the tumors which satisfy the provided threshold value. Although this method results in the loss of samples and thus of tumor classes, it offers a balanced dataset where all the classes are reasonably represented. The results of the application of this technique yielded two datasets of 7,724 and 5,396 samples when thresholds of 300 (top sixteen tumor types) and 450 (top ten tumor types) were used, respectively.

**Fig. 2:**
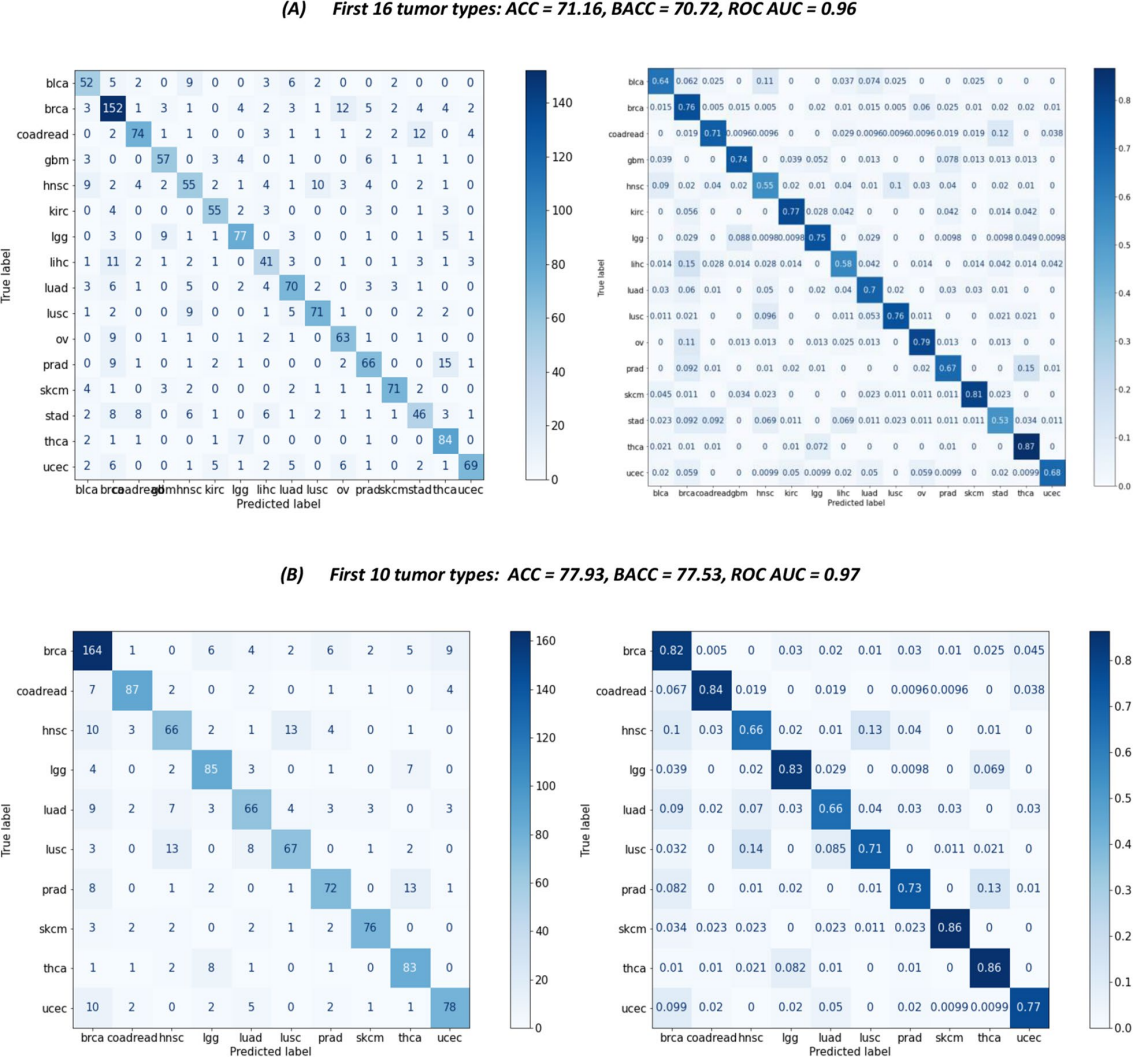
Confusion matrices showing the performance, in terms of accuracy (ACC), balanced accuracy (BACC) and AUC score, of XGBoost model trained with the 16 (**A**) and 10 (**B**) most represented cancer types of the dataset, corresponding to the 70% and 50% of the entire dataset, respectively. The number of calls for somatic point mutations (SPMs) and copy number variations (CNVs) at chromosome arm-level were considered as predictive features of cancer types. This figure shows the two models that achieved the best performance

In the first case (top sixteen tumor types, corresponding to the 70% of the entire dataset), the BACC obtained was 70.72%. As for the AUC, the model yielded 0.96 (Fig. 2A). Regarding the individual tumor accuracy, skcm and thca showed the highest scores (81% and 87%, respectively), while hnsc, lihc, and stad displayed the lowest accuracy score compared to other tumors (< 60%).

As shown in Fig. 2B, the BACC achieved using the top ten tumor types (corresponding to the 50% of the entire dataset) was 77.53% with an AUC score of 0.97. Like the previous model, it was observed that skcm and thca contributed the largest percentage to the overall accuracy score (86%) followed by that of coadread, lgg and brca with approximately 83%, while hnsc and luad accounted for the lowest accuracy score with only 66%.

Overall, in both models, thca tops the table with about 86% accuracy scores, while hnsc, although making up about 5% of the dataset (sixth tumor in terms of number) was found at the bottom of the individual accuracy table.

### Grouping experiments

**Fig. 3:**
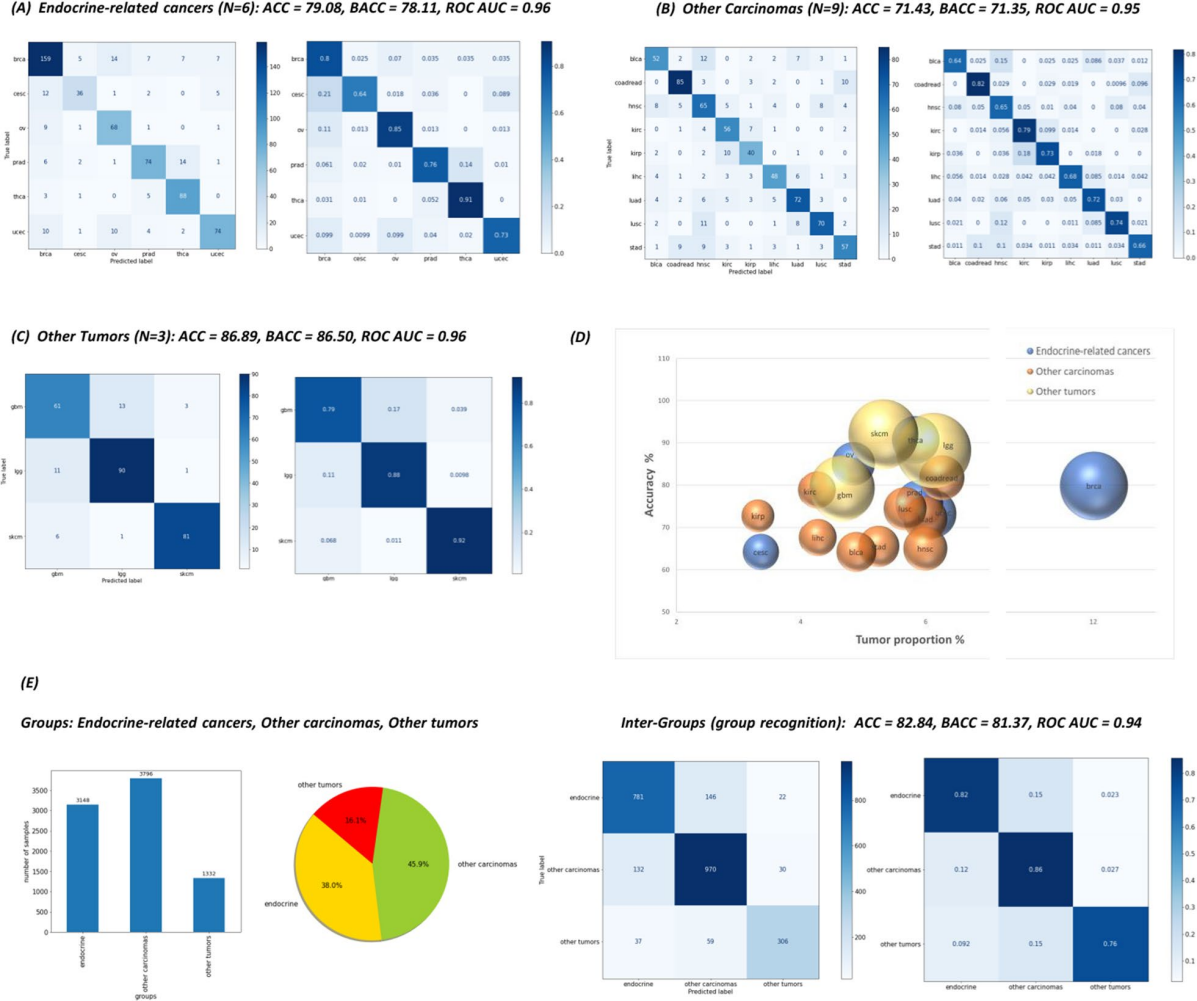
Confusion matrices showing the performance, in terms of accuracy (ACC), balanced accuracy (BACC) and AUC score, of XGBoost models trained with (**A**) Endocrine-related cancers (N = 6: brca, ucec,prad, thca, ov, cesc)), (**B**) Other carcinomas (N = 9: coadread, luad, hnsc, lusc, stad, blca, lihc, kirc, kirp) and (**C**) Other tumors (N = 3: lgg, skcm, gbm) dataset. **(D)** Bubble plot of individual tumor accuracies versus corresponding tumor proportions (number of sample for each tumor type/total number of samples) within the restricted dataset (total of 18 tumor types). Colors indicate the different groups while bubble size corresponds to the individual tumor proportion within the group to which it belongs (number of sample for each tumor type/total number of samples of the corresponding biological group). (**E**) Charts showing the size, in terms of total count and percentage, of each group (Endocrine-related cancers, Other carcinomas and Other tumors) in the new created dataset in which each sample is associated with the group it belonged to, thus with the three groups as targets. The confusion matrix shows the performance of the model

An alternative used approach was to divide the most represented cancer types into groups, based on biological criteria. The different tumor classes were grouped into three distinct groups: endocrine-related cancers, other carcinomas and other tumors, resulting in the generation of three datasets comprising six, nine and three tumors, respectively, for a total of 18 different tumor types (Fig. 1A). These three models achieved 78.11%, 71.35% and 86.50% BACC, respectively, and 0.96 AUC (Fig. 3A-D).

Looking more in detail at the individual accuracy scores, in the endocrine-related cancers dataset, thca and ov achieved the best performance with scores of 91% and 85%, respectively, while the other cancer types within the group, showed accuracy values ranging from 64 to 80%.

Regarding the dataset of other carcinomas, coadread and kirc were the tumors with the highest accuracy scores, with values of approximately 80%.

Other tumor datasets consisted of gbm, lgg and skcm tumors, which showed 79%, 88% and 92% BACC, respectively.

Overall, as with the cut-off threshold experiments described above, we did not observe a direct link between tumor size, in terms of number of samples, and corresponding accuracy scores; for example, although ov was not among tumors with the highest size, this cancer type performed very well (85% accuracy score). On the other hand, ucec was characterized by a high number of samples but an accuracy of only 73%.

After grouping cancers into the three distinct categories, a dataset was produced that unified all the groups but in which each sample was associated with the group it belonged to. As a result, a new dataset with the three categories (endocrine-related cancers, other carcinomas and other tumors) as targets was created.

The bar and pie charts in Fig. 3E show the count and percentage of each target group. Specifically, other carcinomas dataset had the highest count with 3,796 samples, contributing to 45.9% of the new dataset, followed by endocrine-related cancers dataset (3,148 samples, 38%) and other tumors dataset (1,332 samples, 16.1%). Overall, the new model achieved an 81.37% BACC with AUC of 0.94.

Regarding individual accuracy, it was observed that other carcinomas had the highest accuracy score with percentage of 86% followed by endocrine-related cancers with 82% and other tumors with 76%.

Notably, these accuracy values were significantly higher than those obtained by performing random grouping experiments based on the same numerical complexity, thus similar group sizes (Additional file 1**: Figure** S4**.**), highlighting the validity of our biological-based grouping strategy.

Furthermore, in all experiments described, the additional evaluated metrics (sensitivity, specificity, F1-score and MCC) showed consistent results, highlighting the robustness of the models (Additional file 1: Table S5).

## Knowledge extraction phase

XGBoost automatically produces, during the training, a feature ranking of impact on the predicted output, providing a benefit.

**Fig. 4:**
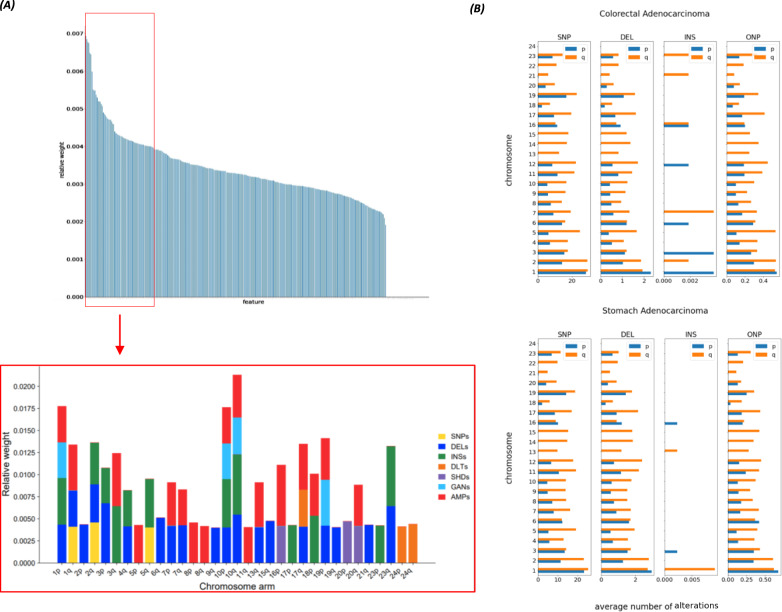
**A** Bar chart showing the complete overview and detail of the features with the greatest importance based on features ranking of impact on the predicted output, automatically produced by XGBoost during the training of the top 16 tumor types. Abbreviations: Single Nucleotide Polymorphisms (SNPs), Deletions (DELs), Insertions (INSs), CNV Deletions (DLTs), Shallow-Deletions (SHDs), Gains (GANs) and Amplifications (AMPs). **B** Comparison of SPMs patterns, thus distribution of alterations at chromosome arm-level, between colorectal adenocarcinoma and stomach adenocarcinoma reported as an example of similarity between tumors at molecular level (cross-cancer similarity) and a possible cause of misclassification errors

Bar chart in Fig. 4A shows the most relevant features using the feature importance functionality of the XGBoost. Among these, alterations in chromosomes 1, 3 and 10 displayed considerable relevance.

We reported feature ranking of cut-off threshold experiment on the top 16 tumor types, chosen as the most representative and, therefore, more interesting as it includes the largest number of different tumor types analyzed. In general, results of feature ranking obtained are basically the same for all trained models (data not shown).

Overall, regardless of the model used, blca, hnsc, lihc and stad represent the tumors with the worst performance, leading to an accuracy always lower than 70%. Furthermore, most errors in the identification of a specific cancer type were observed in tumor pairs such as hnsc and lusc, hnsc and blca, brca and ov, coadread and stad and this may be due to cross-cancer similarities at the molecular level (Fig. 4B).

On the other hand, several tumor types were often misclassified as brca, probably because this represents the tumor characterized by the largest size (994 samples).

## Discussion

Large chromosomal events, such as losses and gains of entire chromosomes, alterations of chromosome arms, copy number variations and rearrangements as well as changes at nucleotide level, including single nucleotide variants and small insertions/deletions, are observed in nearly all cancer genomes [41].

High-throughput sequencing technologies and the resulting increasing number of cancer genome studies have provided an unprecedented opportunity for the understanding of human cancer biology, leading to the identification of genomic features of clinical utility as molecular targets for cancer therapy [42].

In current clinical practice, immunohistochemical analyses are widely used to determine histological type of cancers in both primary and metastatic tumors [43]. However, some tumors are so poorly differentiated that unambiguous immunohistochemical classification is difficult to achieve. Patients with cancer of unknown primary account for about 3–5% of all cases and are typically characterized by poor survival. To date, it is well established that site-specific therapeutic approaches, based on the identification of tumor cells of origin, are more effective than broad-spectrum chemotherapy [44]; thus, determining the cancer type and site of origin is essential to establish the most appropriate treatment strategy [13, 45].

Over the last years, several studies have focused on building mathematical models able to predict cancer type based on molecular characteristics, including gene expression [46–48] and DNA methylation [49, 50] profiles as well as somatic alteration analysis [9, 13, 51].

In this context, ML techniques, lately successfully applied in many fields [52–54], represent a practical instrument to capture complex relationship between molecular alterations and cancer types to build accurate and automated easy-to-use diagnostic tools, and to extract information of biological relevance.

In this study, a new feature extraction method inspired by VSM Computer Science approaches was devised to transform uniformly processed data from TCGA PanCancer Atlas project into a structured dataset for developing a ML model aimed at distinguishing cancer types based on genetic lesions. The method was based on counting the occurrences of molecular alterations (SPMs and CNVs) at chromosome arm-level and at different resolution levels. As previously reported, the combination of SPMs and CNVs can significantly improve the performance of cancer diagnostic tools based on ML approaches [13].

In addition to the heterogeneity of sequencing methods and bioinformatics analysis pipelines, one of the main issues in discriminating multiple cancer types concerns the imbalance in the number of available samples [55].

In our dataset, not all the 32 considered cancer types were equally represented, thus, proper pre-processing techniques were devised to cope with imbalanced data and provide accurate models for as many cancer types as possible.

Overall, by setting a percentage cut-off threshold (top 16 and top 10 tumor types) and grouping the first 18 tumor types based on biological criteria, we obtained models characterized by high reliability and remarkable level of accuracy, ranging from 71 to 86% (AUC 0.94–0.97).

The diagnostic potential of our model (in terms of performance and number of different tumors predicted) is like or higher than models reported in previous studies using ML approaches and based on somatic alterations from whole exome sequencing (WES) data.

In particular, Marquard et al. [56] were able to discriminate among 10 cancer types with 69% accuracy and 6 cancer types with 85% accuracy when only somatic mutations or somatic mutations and copy number alterations were considered, respectively.

By focusing on somatic mutation data, Chen et al. [57] achieved a classification accuracy of 62% in identifying 17 different tumors.

Soh et al. [13], based on the presence of somatic point mutations and copy number alterations in 50 genes as predictive features, achieved approximately 77% accuracy in 28 different cancers.

Compared to WES-based approaches, whole genome sequencing (WGS)-based tumor-type classification showed higher accuracy scores [9, 51, 58]. Jiao et al. [9], achieved an overall accuracy of 91% in discriminating among 24 tumors based on somatic passenger mutations patterns and deep learning approach.

With a similar feature extraction strategy, the model trained by Salvadores et al. [58], distinguished 18 cancer types with 92% accuracy.

By adding driver gene mutations and complex structural variant-related features, Nguyen et al. [51], were able to predict 35 different cancer (sub)types with around 90% accuracy.

Overall, consistent with our findings, all studies of WES and WGS-based classifiers highlighted that model built on multiple features (e.g. point mutations and copy number alterations) outperformed those built on single-type of feature.

To date, although the performance of our model is lower than that obtained by WGS-based studies, the potential use of WGS in clinical practice is still limited due to costs, timing and, most importantly, higher complexity of data analysis.

Furthermore, our classifier offers the great advantage of using a simple preprocessing system; here, a specific feature extraction methodology has been developed to transform the unstructured raw data into a dataset suitable for supervised learning classification tasks, whose simple structure does not necessarily require the adoption of complicated deep learning architectures. As a byproduct, the final dataset has a high level of interpretability with respect to the original data, allowing many statistical analyses.

From a clinical point of view, a practical cancer diagnostic tool should ideally be easy to use in terms of both model building and dataset construction, so that it remains accessible.

Here, we built a simple, accurate and robust model to distinguish tumors, with a large potential clinical applicability related to the model (machine learning *vs* deep learning) and input data (reduced features and simple feature extraction methodology from WES analysis *vs* other WES studies and WGS analysis).

This ML approach could greatly contribute to accurately classifying cancer types, thus enabling personalized treatment strategies. In combination with histopathological examinations, it could potentially address a major challenge in cancer diagnosis and therapy, represented by the classification of tumors of unknown origin.

Moreover, in the scenario of strategies’ integration based on multi-omics data [59, 60], we can also hypothesize that adding different omics datasets as well as merging molecular and pathological data may further improve the performance of our ML model.

The TCGA dataset used in this study contained almost exclusively primary tumor samples, not allowing performance evaluation of our models on metastatic samples of the different cancer types. The only exception was represented by skcm, for which more than 80% of the samples were metastases and the remaining 20% were primary tumors. Interestingly, this cancer type was among the tumors with the highest accuracy scores in all models used.

Therefore, results obtained on skcm suggest that our methodology is experimentally robust and promising in identifying the site of origin starting from metastatic samples, despite the need to test an independent set of metastases.

At the biological level, regardless of strategy used (threshold or grouping models), we observed more easily predictable tumors (such as thca, skcm and ov) and low-performing tumors (such as hnsc, blca, lihc). For example, in both models, skcm and thca maintained high accuracy scores (> 80%), suggesting that the dataset thus constructed is very useful for predicting certain cancer types, while for tumors with lower performance, additional information (features) may be needed to improve the discrimination ability of the model.

As already discussed, previous WES and WGS-based studies [9, 13, 51] as well as our cut-off experiments, have shown that the accuracy on each tumor has a close relationship with the corresponding size and that an imbalance in the dataset makes more difficult to achieve high performance.

Cancer types with the smallest sample size are consistently poorly predicted, thus, in this study we narrowed the analysis up to the top 18 different cancer types.

It is therefore expected that with more training samples available, the accuracy of ML-based diagnostic tools will greatly increase [13].

However, it is evident that some tumors are generally more difficult to be predicted than others, suggesting that accuracy probably also depends on the intrinsic molecular characteristics of the different tumor types, some more heterogeneous than others at molecular level; regardless of size, the individual accuracy scores of certain tumors are in fact relatively low (e.g. ucec, 74%) compared to others (e.g. ov, 85%). As previously reported, ov showed homogenous features at genomic level, while four transcriptional subtypes, three microRNA subtypes and four promoter methylation subtypes were identified [61]. In contrast, four different subtypes, based on genetic mutations and CNVs, have been reported for endometrial carcinoma [62]. This type of tumor also shares genomic features with serous ovarian cancer, the basal-like subtype of breast cancer, and colorectal cancer [62], thus making cross-cancer similarities an additional challenge for its classification.

Therefore, variability in classification accuracy among tumors and misclassification errors could be related to: (i) tumor type heterogeneity and thus the presence of different subtypes, as previously reported for endometrial carcinoma and lung cancer [9, 51]; (ii) a common developmental origin, as observed for uterine and ovarian cancers [50], being both gynecological cancers [63] and (iii) common biological characteristics in tumors of different origin due to cross-cancer similarities [2].

Different cancer types can be divided into a wider range of subtypes and several studies have already highlighted the importance and utility of exploiting molecular data to distinguish them [3–7]. In this context, the availability of more data would allow the training of an updated XGBoost model able to classify additional cancer types and subtypes.

Finally, XGBoost automatically produces a feature ranking of impact on the predicted output, thus providing a benefit in terms of estimation of feature importance from the trained predictive model. One major problem when analyzing this kind of massive data consists of their high dimensionality in terms of large number of features. Feature ranking can both improve data analysis system efficiency and reduce the interference of redundant and irrelevant features [64].

Therefore, through this functionality, the use of XGBoost classifier models also offers the advantage of extracting valuable information of biological interest and obtaining a deeper insight into the most relevant biological processes or characteristics underlying the generated data.

These results represent a springboard towards further insights into the differences and similarities within and between the different cancer types and a deeper interpretation of the biological significance of the most relevant features emerged, steps that may contribute to increasing our understanding of cancer biology.

Overall, we developed an innovative approach which exploits ML techniques for cancer type prediction based on genomic alterations, using one of the largest datasets currently available. Despite the high levels of performance achieved, this study represents the starting point for more advanced analyses. In this context, the integration of other genomic data could further improve the predictive performance of our model, allowing us to classify additional cancer types and subtypes, and shed light on the biological significance of these genomic features.

Radiology and pathology are the main medical fields that extensively tested and used ML-based diagnostic systems over the years [65]. To date, the increasing availability of genomic and epigenomic data represents an additional resource: together with information from medical imaging and clinical data, molecular data can indeed guide the implementation of personalized medicine and improve the prediction performance of ML-based diagnostic tools [66]. For global high-impact diseases, such as cancer but also cardiovascular and neurological disorders, the complexity of the genomic landscape and the underlying molecular mechanisms is a major limitation to the development of accurate tools for early diagnosis and effective treatment [66]. Overall, we can therefore speculate on the potential applicability and utility of genomics-based ML approaches like ours, to other multifactorial diseases as well.

## Conclusions

A boosted and accurate ML model able to discriminate among different cancer types based on somatic mutations and copy number alterations was developed. In combination with histopathological examinations, this approach could have potential clinical application in terms of cancer diagnosis improvement. Further analyses, adding more features and/or using a larger number of samples as well as dividing tumor into the main molecular subtypes, could increase the performance of the classifier and extract information of biological relevance.

An interesting and promising perspective is also represented by the possibility of accurately determining tumor site of origin by analyzing cell-free tumor DNA (cfDNA). Given the continuous increase of sensitivity and cost-effectiveness of next-generation sequencing technologies, there are realistic prospects to apply ML approaches to cfDNA analysis to early detect cancers [67].

### Supplementary Information

**Additional file 1: Figure S1.** Example of a SPM[*t*] dataset for a generic tumor type *t.* **Figure S2.** Example of a CNV[*t*] dataset for a generic tumor type *t*. **Figure S3.** Pseudocode of the VSM data transformation procedure. **Figure S4**. Charts showing the size, in terms of total count and percentage, of each random group in the newly created dataset with groups as targets and confusion matrix showing the performance [accuracy (ACC), balanced accuracy (BACC) and AUC score] of the model; hyperparameters are also reported. Of note, accuracy values obtained from random grouping experiments reported here, were significantly lower than those obtained by performing grouping experiments based on biological criteria and characterized by the same numerical complexity (similar group sizes).

## Data Availability

Publicly available datasets analyzed in this study (TCGA PanCancer Atlas Studies) are available in the cBioportal repository (https://www.cbioportal.org/). The datasets generated during the current study are available from the corresponding author on reasonable request.

## Abbreviations

Balanced Accuracy: BACC
Bladder urothelial carcinoma: Blca
Brain lower grade glioma: Lgg
Breast invasive carcinoma: Brca
Cell-free tumor DNA: CfDNA
Cervical squamous cell carcinoma: Cesc
Colorectal adenocarcinoma: Coadbread
Copy Number Variations: CNVs
Deletions: DELs
Glioblastoma multiforme: Gbm
Head and neck squamous cell carcinoma: Hnsc
Insertions: INSs
Kidney renal clear cell carcinoma: Kirc
Kidney renal papillary cell carcinoma: Kirp
Liver hepatocellular carcinoma: Lihc
Lung adenocarcinoma: Luad
Lung squamous cell carcinoma: Lusc
Machine Learning: ML
Natural Language Processing: NLP
Next Generation Sequencing: NGS
Oligo Nucleotide Polymorphisms: ONPs
Ovrian serous cystadenocarcinoma: Ov
Prostate adenocarcinoma: Prad
Sarcoma: Sarc
Single Nucleotide Polymorphisms: SNPs
Skin cutaneous melanoma: Skcm
Somatic Point Mutations: SPMs
Stomach adenocarcinoma: Stad
The Cancer Genome Atlas: TCGA
Thyroid carcinoma: Thca
Uterine corpus endometrial carcinoma: Ucec
Vector Space Model: VSM
Whole Exome Sequencing: WES
Whole Genome Sequencing: WGS
