# Supplementary figures and images for: Classification of tumor types using XGBoost machine learning model: a vector space transformation of genomic alterations
Source: J Transl Med. 2023 Nov 21;21:836. doi: 10.1186/s12967-023-04720-4 (PMC10664515; doi:10.1186/s12967-023-04720-4)

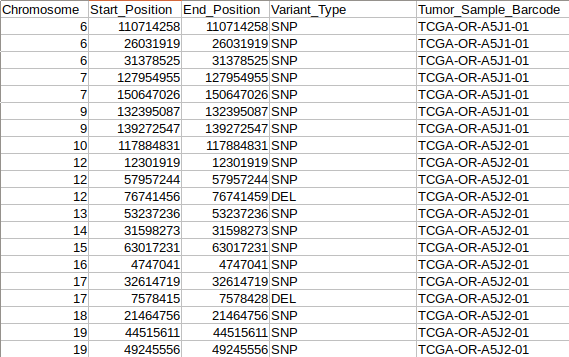

Supplement: Supplementary file 1 — Additional file 1: Figure S1. Example of a SPM[t] dataset for a generic tumor type t. Figure S2. Example of a CNV[t] dataset for a generic tumor type t. Figure S3. Pseudocode of the VSM data transformation procedure. Figure S4. Charts showing the size, in terms of total count and percentage, of each random group in the newly created dataset with groups as targets and confusion matrix showing the performance [accuracy (ACC), balanced accuracy (BACC) and AUC score] of the model; hyperparameters are also reported. Of note, accuracy values obtained from random grouping experiments reported here, were significantly lower than those obtained by performing grouping experiments based on biological criteria and characterized by the same numerical complexity (similar group sizes). [file 12967_2023_4720_MOESM1_ESM.zip › ESM/Additional file 1_Figure_S1.png]

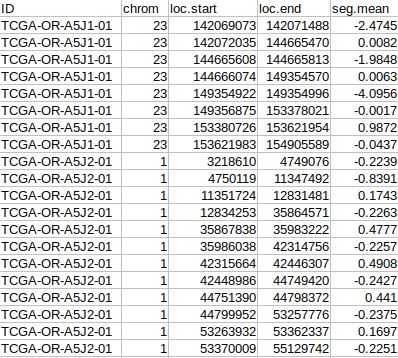

Supplement: Supplementary file 1 — Additional file 1: Figure S1. Example of a SPM[t] dataset for a generic tumor type t. Figure S2. Example of a CNV[t] dataset for a generic tumor type t. Figure S3. Pseudocode of the VSM data transformation procedure. Figure S4. Charts showing the size, in terms of total count and percentage, of each random group in the newly created dataset with groups as targets and confusion matrix showing the performance [accuracy (ACC), balanced accuracy (BACC) and AUC score] of the model; hyperparameters are also reported. Of note, accuracy values obtained from random grouping experiments reported here, were significantly lower than those obtained by performing grouping experiments based on biological criteria and characterized by the same numerical complexity (similar group sizes). [file 12967_2023_4720_MOESM1_ESM.zip › ESM/Additional file 1_Figure_S2.png]
